# Supplementary material for: Deep learning-based age estimation from clinical Computed Tomography image data of the thorax and abdomen in the adult population
Source: PLoS One. 2023 Nov 7;18(11):e0292993. doi: 10.1371/journal.pone.0292993 (PMC10629654; doi:10.1371/journal.pone.0292993)
Supplement: Attachment — Submitted filename: Response to Reviewers.docx [file pone.0292993.s001.docx]

Dear Prof. Homolka, dear Reviewers,

we appreciate the opportunity to submit our revised manuscript (PONE-D-23-10836) titled

*“Deep learning-based age estimation from clinical Computed Tomography image data of the thorax and abdomen in the adult population”*

Bjarne Kerber, Tobias Hepp, Thomas Küstner, Sergios Gatidis.

We thank you for taking the time to edit and review our work and are grateful for the insightful comments. We addressed the concerns raised in the decision letter and incorporated the suggestions into our revised manuscript. Please find our response below.

Yours sincerely

The authors

Response

# Editor Comments and Responses

- **Comment 1**: *As also stated by Reviewer #1, the limitations should more clearly be concentrated in a subsection at the end of the discussion.*

**Response:** We thank the Editor for pointing out, that the limitations should be written more concisely. We improved our manuscript accordingly.

- **Comment 2:** *Age estimation is most demanding but likely most useful for minors under and about the age of 20. If these are excluded, this is a strict limitation of the study that needs to be reflected in the title, because many potential readers will be interested in the age determination of younger persons, e.g. for forensic issues. So a more appropriate title would be, e,g, "Deep learning-based age estimation from clinical Computed Tomography image data of the thorax and abdomen in the adult population“*

**Response:** The editor is right, that this distinction should be made to avoid confusion. Unfortunately, we had no access to a sufficient number of pediatric cases. The revised manuscript was given a more appropriate title.

- **Comment 3:** *Regarding the usually performed and necessary validation steps refer to Recht, M. P. et al. Integrating artificial intelligence into the clinical practice of radiology: challenges and recommendations. Eur Radiol 30, 3576-3584, doi:10.1007/s00330-020-06672-5 (2020), Technical validation section, and make sure to verify robustness, reproducibility, and generalizability appropriately. This does not seem possible with the limited data set used for verification. This needs to be done appropriately, especially robustness and generalizability. This needs to be checked and discussed.*

**Response:** We are grateful that the editor has addressed this important point. The literature cited by the editor provides valuable recommendations for the technical validation of machine learning models to be used in healthcare with respect to robustness, reproducibility, and generalizability. We addressed these challenges by conducting additional experiments and incorporating the obtained results in our revised manuscript. In particular, we performed two analyses regarding variation in age estimation. First, we processed the images from the test dataset using perturbation methods such as noise injection, random resizing and random intensity shifts and compared the age predictions to the predictions for the original images. Second, for individuals in the test set with multiple scans, we tested the reproducibility of the age prediction of our model for different scans. We found that our trained model, in addition to generalizing well to unseen data, has high overall robustness and reproducibility. The relatively large size of our test data set allowed us to conduct these additional experiments.

# Reviewer #1

## Abstract

- **Comment 1:** *"Recently, it has been shown that accurate chronological age prediction is possible using deep learning-based approaches trained on healthy cohorts." Please be more specific about the modality of this approach, It sounds like your study has already been conducted.*

**Response:** Thank you for raising this point. The reviewer is right, that this needs clarification. We have revised the corresponding section accordingly.

## Introduction

- **Comment 2:** *l42-44: This sentence does not fit and could be removed/merged with the next paragraph.*

**Response:** Agree. We have revised the manuscript accordingly.

- **Comment 3:** *l57: there is a gap since deep learning as such was not mentioned/introduced.*

**Response:** Thank you. We have revised the manuscript accordingly.

- **Comment 4:** *l57-66: these paragraphs feel somehow disconnected.*

**Response:** Agree. We have revised the manuscript accordingly.

- **Comment 5:** *l86: please give references for MR+age estimation.*

**Response:** Thank you for pointing that out. We included the requested references.

## Methods

- **Comment 6:** *l106-109: I am unsure if "rule out significant trauma" is correct. Please explain. Also, does this mean that you assumed that emergency room patients are (otherwise) healthy? If so, please be more specific about it, already in the Abstract.*

**Response:** Thank you for your valuable suggestion. The reviewer is right, that this needs clarification. We will improve our manuscript in this regard. We included emergency room patients, who received a trauma CT where no pathological findings were reported into our work.

- **Comment 7:** *l109-110: Why were patients <20 or >85 years excluded? I would have expected <18 years or maybe <21 years-- and no exclusion of older adults.*

**Response:** Thank you for raising this important point. In the initial dataset, there were only small numbers of patients younger than 20 years and older than 85 years, so that we restricted the age range to the subjects, where we had a sufficient number of examples.

- **Comment 8:** *l111: Please give a patient flowchart including ALL train and test cohorts exclusion criteria. E.g., were multiple images of the same patient excluded?*

**Response:** Thank you for this valuable comment. We will clarify on the issue. Only a single study for each patient was included. The requested flowchart was included in our revised manuscript.

- **Comment 9:** *l130: 3mm seems large, was this part of the inclusion criteria? What was the inplane resolution?*

**Response:** Thank you for this question. Examinations were drawn from the time period between 2010 and 2019, were this was a standard slice thickness for emergency trauma scans in our institution.

- **Comment 10:** *l146: This is 'critical' since this preprocessing converts the 3D data into 2D so that it could lose information. Since it is now virtually a set of localizers, it would be reasonable to compare your model to existing 2D models in the Introduction section. Please also add an image with, say, three examples to get a visual impression of the projections. It is part of the visualization, but a dedicated figure upfront might help.*

**Response:** Thank you for raising an important point here. We chose this approach to make model training and predictions more computationally feasible, while still preserving some of the 3D-information. We referenced 2D-approaches in the introduction (Langner and Wikström, Raghu and Weiss) and compared them in the discussion. We added the requested input examples to our revised manuscript. We agree that using a 3D-model could potentially improve CT-based age estimation. Overcoming associated challenges regarding algorithm design and computational feasibility will be part of future research.

- **Comment 11:** *l149: Why offline augmentations were performed instead of the very common online augmentations?*

**Response:** Thank you for this question. We used offline data augmentation to save training time and avoid CPU-bottlenecks. However, the wording is misleading. It was meant, that data augmentation was performed *for* the training process, not *during* it. We clarified the statement in the revised manuscript.

- **Comment 12:** *l151: I need clarification about the network. I thought the 2D shape of 224x224 was chosen to fit the default ImageNet resolution, yet, you state that you cropped 56x112 pixels and resized them between 1 and 1.5. This resizing would result in images of different sizes between 56x112 and around 85x172? So the network was fed with different sizes? Or was this resized to 224x224? If so, it seems to be a very small crop size?*

**Response:** Thank you for raising this point. Every input image had the standard ImageNet resolution of 224x224 pixel. The cropped patches were randomly resized, shifted and rotated, while the rest of the image was filled with zeros. An example is shown in Figure 2.

- **Comment 13***: l154-166: Personally, I'm not too fond of splitting the description of network architecture and implementation details. Please merge the relevant sections (2.4,2.5,2.7) into a single section, explaining all parameters needed for reproducing the network. Add hardware and software details at the end of this paragraph or in an extra section before statistical analysis. Then continue with 2.6, which can be regarded as post-processing. About the loss: Is the given loss function the only loss function used for training?*

**Response:** Agree. We revised our manuscript accordingly. The loss function mentioned is the only loss function used for training.

- **Comment 14***: l185: There is no mention that the splitting was performed along patients instead of images?*

**Response:** Thank you for this question. The splitting was performed along patients. This is now mentioned in the revised manuscript.

- **Comment 15:** *l192: I misread this at first; please state clearly that you do not retrain but re-use the trained models for training, i.e., you create a small ensemble.*

**Response:** Thank you for raising this point. We trained a new model for each fold of the cross-validation. The predictions on the validation datasets were single predictions from the trained model of said validation fold. The predictions on the previously unseen test dataset were average predictions of the five trained models. We will explicitly state that in our revised manuscript.

## Results

- **Comment 16:** *l201: I am missing relative errors. While it is customary to only report MAEs in a machine learning context, in the medical context, a relative error is more reasonable since, say, a difference of 10 years might be different for an 18 year-old than for a 85 year-old. Please compute the relative errors and also plots them in a suitable way.*

**Response:** Thank you for your suggestion. In response, we added a graph showing that there is no monotonic relation between chronological age and MAE. Therefore relative errors would only be of limited value.

- **Comment 17***: l214: Subanalyses, especially one wrt kernels and scanners, were not mentioned before. Could you add more details to the Methods part?*

**Response:** Agree. We revised our manuscript accordingly.

- **Comment 18:** *l222: There is something wrong with the stated prediction error. 6.24 maybe?*

**Response:** Agree. We revised our manuscript accordingly.

- **Comment 19:** *l230-235: These paragraphs feel very disconnected. Please rewrite. Maybe it can help to put all results into a table and compute them for CV and test?*

**Response:** Thank you for this suggestion. We revised the mentioned paragraph.

- **Comment 20:** *l241: "than in the ... validation dataset?". This isn't very clear (also in the plots) since you actually never split off a validation set but used only CV.*

**Response:** Thank you for raising this point. We clarified this sentence in our revised manuscript.

- **Comment 21***: Figure 3: so the network never predicted age <20 years and >81 years? This seems strange; can you explain?*

**Response:** Thank you for this valuable question. We chose the gaussian negative log likelihood loss as loss function, where the network predicts the mean and standard deviation of a gaussian distribution, in which the true age should lie. This could be a possible explanation for this behavior, because the network has a tendency to avoid extreme values.

## Discussion:

- **Comment 22:** *l283: I do not think your MAE is competitive with the UK biobank study. The R2 is, but what does a linearly measured correlation tell us about prediction quality?*

**Response:** Thank you for this very relevant question. We agree and revised our manuscript accordingly. The core difference between the two works is the much larger age span with a higher standard deviation of our study population in comparison to the UK Biobank study. The significant R^2^ measured the correlation between the predicted age and the true age, which is supposed to be a linear relationship. The R^2^ was chosen to show, that the predictions of our model are strongly and significantly correlated to the true age, which shows the quality of the predictions of our model. We incorporated a Bland-Altman-plot of the network predictions and true age to underpin the prediction quality visually.

- **Comment 23:** *l290: Why is the population different? The UK Biobank should have 'random' patients-- isn't this the same as in the emergency room?*

**Response:** Thank you for raising this interesting point. The study population was chosen from the emergency room, because a large number of patients received a non-pathological trauma scan. The population of the emergency room differs from the general population, because e.g. more young people are involved in car accidents, while older people are more likely to suffer trauma from falls, which also leads to a certain selection of individuals, while the UK Biobank study is a true population study.

- **Comment 24:** *l295-299: I think you mean "body regions", however, I find this paragraph hard to follow. Besides, could your uncertainty be a statistical effect of cutting off patients <20 years (the same for >85 years)?*

**Response:** Thank you for this suggestion. The reviewer is right, that the paragraph needs to be worded better.

We don’t believe that cutting off patients would influence the uncertainty. There was a medium strong correlation between the number of original training examples and uncertainty, which might indicate, that the model uncertainty reduces, when more training images have been seen. The uncertainty on the “edges” of our age distribution is lower than in the middle, because there are plenty of training examples, while there are less examples around the mean of our age distribution.

- **Comment 25:** *l305: Can you cite other studies with a more homogenous population? Both studies from above should also be very heterogeneous.*

**Response:** Thank you for raising this point. The UK Biobank was in this respect more homogenous, that the same, standardized imaging protocol was used and subjects were preselected, while we used a more clinically realistic dataset of actual patients.

- **Comment 26:** *l324: This could maybe have been avoided by normalized wrt pixel/voxel size.*

**Response:** Thank you for this suggestion. We suspect that the inclusion of the legs changed the image data and made important regions smaller, but we chose to include said images anyways to make the model more robust and also show its robustness.

## Code/Data

- **Comment 27:** *Can you share the code and, if possible, also the model? Since the test data is freely available, this would help with reproducibility.*

**Response:** Agree. We will make the requested data available. The code for preprocessing, the model, its weights and the Score-CAM implementation can be found at <https://github.com/BjarneKerber/age_estimation>.

# Reviewer #2

## Major comments

- **Comment 1:** *Interpretation of grad-cam heatmaps has been called into question in recent studies. Would dial back claims that score-cam can improve interpretability/identify specific features important for age estimation. Also, it’s unclear whether interpretability analyses were done systematically. Did you quantify how often certain regions of the body were highlighted by these heatmaps? If not, would make it clear that the interpretability results are a qualitative assessment by the study authors and not a rigorous investigation.*

**Response:** Thank you for this important comment. We made clear, that the interpretability results are just qualitative to avoid confusion. We revised our manuscript accordingly.

- **Comment 2:** *The authors should make their code and model weights publicly available to facilitate future research. It would also be helpful for the authors to make their predicted ages for the training/testing datasets publicly available and any other derived data as far as possible.*

**Response:** Thank you for your comment. We will make the requested data available. The code for preprocessing, the model, its weights and the Score-CAM implementation can be found at <https://github.com/BjarneKerber/age_estimation>.

- **Comment 3:** *The publicly available testing dataset is not adequately described. Who are these individuals? Why were they getting CT? Did they have any characteristic information available? A Table 1 describing the two cohorts would be very helpful to better interpret the results of the study.*

**Response:** Thank you for your suggestion. We added additional information about the dataset in our manuscript. The whole dataset is described in detail *“A whole-body FDG-PET/CT Dataset with manually annotated Tumor Lesions. Sci Data. 2022 Oct 4;9(1):601. doi: 10.1038/s41597-022-01718-3. PMID: 36195599; PMCID: PMC9532417”*.

- **Comment 4:** *Please clarify the limitations of the study in the Discussion. Would include things like*
  a. *Small sample size for training to develop a deep learning model*
  b. *Unclear demographic characteristics of these cohorts – will need to evaluate on a more diverse cohort*
  c. *Unclear clinical applicability, how specifically will clinicians/researchers use this model?*
  d. *Single center, retrospective study – needs to be evaluated prospectively*
  e. *Unclear comorbidity history and how this impacts age estimates*

**Response:** Thank you for your valuable suggestions. We incorporated the requested points into the limitations section of our revised manuscript.

## Abstract

- **Comment 5:** *The authors state that aging “… leading to morphological change that can be assessed on medical imaging such as computed tomography scans” – have others shown this already? If so, please highlight such studies in the discussion and potentially compare your model to these existing models. If not, then this is the hypothesis of your work and not a statement of the background.*

**Response:** Thank you for pointing this out. It is a scientific consensus, that CT image data is able to reflect the morphological change occurring during the aging process. In our work we want to show, that automated age estimation is possible using said fact. To our knowledge, no models exist for automated age estimation on thorax/abdomen CT image data generated in a clinical context. We clarified this statement in the revised manuscript.

## Methods

- **Comment 6:** *Why wasn’t data augmentation performed online? Why were 49 augmented images chosen? Did this number impact generalization performance/training time?*

**Response:** Thank you for this question. Offline data augmentation saved training time and helped to avoid CPU-bottlenecks. The number of augmented images was chosen empirically, because even the small network used needed strong regularization, which we wanted to achieve by using extensive data augmentation.

- **Comment 7:** *Small typo – ”ResNet18-architecture pretrained on the by Pytorch”*

**Response:** Thank you for pointing this out. We fixed the typo.

## Results

- **Comment 8:** *The first line of the results is a little unclear to me – “achieved MAE after 5-fold cross validation” – is this the average MAE across the 5 validation folds? Or is this the result on the independent testing data? Would be helpful to define mean prediction error and mean absolute error.*

**Response:** Thank you for your suggestion. You are right, the computed MAE is the average across the 5 validation folds. We improved the wording in the referenced section.

- **Comment 9:** *Typo in the comparison of prediction error between female and male subjects (-3.57 +/- 8.09 vs. 62.46)*

**Response:** Thank you for pointing this out. We fixed the typo.

- **Comment 10:** *In the uncertainty estimation paragraph, I think the result comparing uncertainty vs. prediction error is the most important and should be better highlighted.*

**Response:** Thank you for this proposal. We revised our manuscript accordingly.

## Discussion

- **Comment 11:** *Wouldn’t compare these results with study focusing on whole-body MRI data since there is a substantially different N and different modality here.*

**Response:** The reviewer is right, that the mentioned study was able to use a much more extensive database and that the modality influences prediction quality, because e.g. MRI has a better soft tissue contrast. We chose to compare our work with this study, because the approach is similar and discussed the differences you correctly mentioned. We furthermore compared the results with the age estimation on the CheXpert dataset, because there was also a similar approach used.

- **Comment 12:** *Have any others attempted to estimate age from CT? This will be good to highlight instead*

**Response:** Thank you for your suggestion. To our knowledge, this is the first study to use thorax/abdomen CT image data for automated age estimation in the adult population. We thus compare our results with other studies using MRI or radiography data.

- **Comment 13:** *Do you think the higher estimated uncertainty in the test set was due to generalization error or due to differences in patient characteristics? What were the differences in patient characteristics between the two populations?*

**Response:** Thank you for asking this very relevant question. There was a higher MAE on the test dataset. The estimated uncertainty had a significant positive correlation with the MAE on both the training and validation and on the test dataset. We intended the estimated uncertainty to act as an indicator of possible higher prediction error. The uncertainty is probably influenced by differing characteristics between the training and the test dataset, that also influenced the generalization error. The differences in patient characteristics are summarized in the discussion and include a different mean age, a higher percentage of female subjects as well as a different indication for performing a CT-scan, namely the suspicion of malignancy.

- **Comment 14:** *Any breakdown available of reasons CTs were performed in the testing dataset?*

**Response:** Thank you for this question. CTs in the test dataset are derived from PET/CT examinations, which were performed because of suspected malignant tumors. The whole dataset is described in detail in *“A whole-body FDG-PET/CT Dataset with manually annotated Tumor Lesions. Sci Data. 2022 Oct 4;9(1):601. doi: 10.1038/s41597-022-01718-3. PMID: 36195599; PMCID: PMC9532417”*.

- **Comment 15:** *Again, would dial back claims about “features” being used for age prediction*

**Response:** Agree, we revised our manuscript accordingly.

- **Comment 16:** *You state in the discussion that uncertainty “indicated the possibility of higher prediction errors as it was intended to” – These results were not clearly stated – did uncertainty correlate with error?*

**Response:** Thank you for this interesting question. There was a weak, but significant correlation between uncertainty and absolute prediction error (l.269). The relationship between prediction error and uncertainty is also shown in Fig. 4.
